# Comparative Evaluation of Quantitative Real-Time PCR and a Laboratory-Developed Chip-Based Real-Time Digital PCR for *JAK2* V617F Allele Burden in Myeloproliferative Neoplasms

**DOI:** 10.3390/diagnostics16152333

**Published:** 2026-07-25

**Authors:** Sunggyun Park, Kyoungbo Kim, Jung-Sook Ha

**Affiliations:** Department of Laboratory Medicine, Keimyung University School of Medicine, Daegu 42601, Republic of Korea; nosdolu1@gmail.com (S.P.); kimbo707@dsmc.or.kr (K.K.)

**Keywords:** digital PCR, chip-based digital PCR, myeloproliferative neoplasm, *JAK2*

## Abstract

**Background:** Precise quantification of *JAK2* V617F variant allele frequency (VAF) is clinically important in Philadelphia chromosome-negative myeloproliferative neoplasms (MPNs). This study evaluated the analytical performance and clinical utility of a laboratory-developed chip-based real-time digital PCR (dPCR) assay for *JAK2* V617F and compared it with a commercial quantitative real-time PCR (qPCR) assay. **Methods:** Residual DNA extracts from 76 *JAK2* V617F-positive clinical specimens collected from patients with suspected MPNs were analyzed using both qPCR and dPCR. Analytical performance was assessed according to CLSI-based approaches, including limit of blank (LoB), limit of detection (LoD), precision, linearity, and method comparison. Associations between VAF and hematologic parameters, as well as diagnostic groups, were also examined. **Results:** Both methods showed a LoB of 0. The LoD was 0.1167% for qPCR and 0.0846% for dPCR, and dPCR demonstrated lower variability than qPCR across high-, intermediate-, and low-concentration samples, with the largest difference observed at low VAF levels. Linearity was excellent for both assays (R^2^ = 0.988 for qPCR and 0.999 for dPCR), although dPCR showed less low-concentration bias. The two methods were highly correlated (Pearson r = 0.9789, R^2^ = 0.958), but dPCR yielded slightly higher VAF values overall. VAF was significantly higher in polycythemia vera than in essential thrombocythemia, and both qPCR- and dPCR-based VAFs were positively correlated with white blood cell count. **Conclusions:** The chip-based laboratory-developed dPCR assay showed high concordance with qPCR while providing lower LoD and better precision at low allele burden. These findings support its potential utility as a sensitive alternative for *JAK2* V617F quantification in clinical laboratories, particularly in settings requiring accurate low-level detection and follow-up monitoring.

## 1. Introduction

Philadelphia chromosome-negative (Ph−) myeloproliferative neoplasms (MPNs) are clonal hematopoietic disorders arising from the malignant transformation of multipotent hematopoietic stem cells and include polycythemia vera (PV), essential thrombocythemia (ET), and primary myelofibrosis (PMF) [1,2]. *JAK2* V617F is the most common somatic mutation in these disorders, being detected in approximately 95% of patients with PV and 50–60% of those with ET or PMF, and the 2022 International Consensus Classification (ICC) strongly recommends the use of highly sensitive molecular assays capable of detecting variant allele frequency (VAF) below 1% for accurate diagnosis and subclassification of MPNs [2,3]. The *JAK2* V617F allele burden is closely associated with disease phenotype and prognosis; a higher allele burden has been linked to an increased risk of thrombotic events and progression to myelofibrosis, whereas a lower allele burden in PMF has been associated with inferior survival and an increased risk of leukemic transformation [4,5,6,7]. Because of this clinical significance, precise quantification of the *JAK2* V617F allele burden has emerged as an important tool for diagnosis, therapeutic monitoring, and prognostic assessment [6,8].

In addition, the *JAK2* V617F allele burden is directly associated with the hematologic phenotype of patients with MPNs. Previous studies have shown significant correlations between *JAK2* V617F allele burden and white blood cell count (WBC), hemoglobin (Hb), red blood cell count (RBC), and platelet count (PLT) [8,9]. In particular, a high allele burden in PV has been associated with more marked erythrocytosis and increased thrombotic risk, whereas a relatively low allele burden (≤50%) is typically observed in ET [4,7,8]. Thus, allele burden serves not only as a marker of mutation detection but also as an important quantitative biomarker for MPN subclassification and assessment of disease severity [8,9,10].

At present, quantitative real-time PCR (qPCR) is the most widely used method for detecting *JAK2* V617F in clinical laboratories, but it has methodological limitations, including strong dependence on external standard curves and substantial inter-laboratory variability [11,12,13]. As an alternative, digital PCR (dPCR) has attracted increasing attention because it partitions nucleic acid samples into thousands to tens of thousands of independent reaction compartments, thereby enabling absolute quantification at the single-molecule level [14,15,16]. By directly counting DNA molecules based on Poisson statistics without the need for an external standard curve, dPCR provides highly reproducible quantitative results. Various dPCR platforms, including droplet digital PCR (ddPCR), have shown high concordance with qPCR while providing superior analytical sensitivity, particularly in terms of the limit of detection (LoD) [12]. The utility of dPCR has been especially emphasized in clinical settings that require highly sensitive quantification, such as minimal residual disease (MRD) monitoring during JAK inhibitor therapy [6,12].

More recently, some chip-based digital PCR platforms have been designed to collect amplification curves from individual partitions in real time, thereby combining the absolute quantification capability of dPCR with the amplification-curve interpretability of qPCR [16,17,18,19]. However, few studies have simultaneously reported a systematic analytical performance evaluation of such real-time dPCR-based laboratory-developed tests (LDTs) and a direct comparison with a commercial qPCR assay [12,18,20]. Therefore, in the present study, an LDT dPCR assay performance was established using residual clinical specimens from MPN patients in accordance with CLSI guidelines, and evaluated through a method comparison with commercial qPCR.

## 2. Materials and Methods

### 2.1. Study Subjects and Samples

This study initially included 94 residual DNA extracts obtained from specimens that had tested positive by clinically performed qualitative *JAK2* V617F PCR in patients with suspected MPN between January 2018 and June 2022 at the clinical laboratory of Keimyung University Dongsan Hospital, Daegu, Republic of Korea. For quantitative method comparison between qPCR and dPCR, only specimens that yielded valid quantitative results on both platforms were included, resulting in a final comparison set of 76 clinical specimens, of which 16 were derived from bone marrow (BM) samples and 60 from peripheral blood (PB) samples. The residual DNA extracts were used for quantitative analysis of *JAK2* V617F variant allele frequency (VAF) by qPCR and laboratory-developed test digital PCR (LDT dPCR).

### 2.2. Quantitative Real-Time PCR for JAK2 V617F

Quantitative analysis of *JAK2* V617F was performed using the Bio-Rad CFX96 real-time PCR system (Bio-Rad Laboratories, Hercules, CA, USA) and the Real-Q *JAK2* V617F Quantification Kit (Biosewoom, Seoul, Republic of Korea). All experimental procedures were conducted according to the manufacturer’s instructions for use (IFU). The assay was performed in a 96-well plate format, with wild-type (WT) DNA and *JAK2* V617F mutant DNA measured independently in separate wells. Fluorescence signals were detected in a single FAM channel. Sample DNA concentrations were initially measured using a NanoDrop spectrophotometer and then normalized to 5 ng/μL genomic DNA by dilution with distilled water. Five microliters of each sample DNA were added to each reaction, corresponding to 25 ng of genomic DNA per reaction. The PCR reaction mixture consisted of 5 μL of sample DNA and 20 μL of master mix, yielding a total reaction volume of 25 μL. For absolute quantification, four plasmid DNA standards included in the kit were run in parallel in each experiment. The standard concentrations were 2 × 10^6^, 2 × 10^4^, 2 × 10^3^, and 2 × 10^2^ copies, and separate standard curves were generated for the WT and mutant assays. The detailed PCR protocol is listed in the Supplementary Materials. VAF was calculated using copy numbers derived from the WT and mutant reactions according to the following formula: VAF (%) = mutation copies/(mutation copies + wild-type copies) × 100.

### 2.3. Chip-Based Real-Time Digital PCR Assay for JAK2 V617F

The LDT dPCR assay was performed using the LOAA system (Optolane, Seongnam, Republic of Korea), a chip-based real-time digital PCR platform. This instrument is a cartridge-based modular system in which cartridges operate independently, allowing the number of simultaneously running cartridges to be adjusted according to the purpose of the experiment and the required throughput. In the present study, a configuration capable of analyzing up to eight cartridges simultaneously was used. Each reaction was performed using a blank Dr.PCR 20K Cartridge-PF (Cat. No. CTR002-R; Optolane) for research use only (RUO), into which the investigator-prepared reaction mixture and sample were loaded.

Primers and probes for *JAK2* V617F detection were designed to generate a 92 bp amplicon, including the c.1849G(V617) position, based on the *JAK2* reference sequence NM_001322194.2. Each reaction contained a total of 25 ng genomic DNA, the same amount used in the qPCR experiment. The detailed primer and probe sequence and PCR protocol are listed in the Supplementary Materials.

After amplification, raw data generated by the LOAA system were transferred to a PC and analyzed using OnPointPro software version 1.1.9 (Optolane). For each reaction, fluorescence scattergrams and real-time amplification curves were reviewed to confirm that partition-level positive/negative calling had been appropriately performed in both the wild-type and mutant channels. The software performed absolute quantification by accounting for the fraction corresponding to 5 μL of template DNA (25 ng genomic DNA) within the final 30 μL cartridge reaction volume and reported copy numbers for wild-type and mutant targets separately (Supplementary Materials, Figure S1). In this study, the final copy numbers reported by the software were used without further adjustment. VAF was calculated in the same manner as for qPCR. All specimens were analyzed once without replicate testing.

### 2.4. Analytical Performance Evaluation

The analytical performance study was performed by constructing an in-house evaluation panel using a high-burden positive patient sample. After identifying a patient specimen with an initial *JAK2* V617F allele burden of approximately 90%, mixed panels with different expected VAF levels were prepared by combining this specimen with a *JAK2* V617F-negative normal sample at predefined ratios. These in-house prepared panels were used for both LoD and linearity evaluation (Table 1).

For linearity assessment, serial dilutions of the approximately 90% VAF sample were prepared to generate seven levels (Level 1–Level 7) with expected VAFs of 90% (undiluted), 60%, 30%, 10%, 5%, 3%, and 1%. Expected VAFs were derived from the mixing ratios, and detailed preparation ratios and expected VAF values are provided in Table 1. For LoD assessment, an approximately 1% VAF mixed sample (Level 7) was further subjected to serial two-fold dilution to prepare 10 dilution levels (LoD 1–LoD 10), from which concentrations suitable for probit analysis were selected for the final experiments.

Detection capability was evaluated sequentially in terms of limit of blank (LoB) and limit of detection (LoD) according to CLSI EP17-A2 guidelines [21]. LoB evaluation was intended to estimate the upper limit of values expected from repeated measurements of blank specimens, and LoD evaluation was intended to estimate the lowest concentration at which the analyte could be classified as positive based on hit-rate data from low-concentration specimens, usually corresponding to a 95% detection probability.

For LoB evaluation, the approach described in EP17-A2 for molecular measurement procedures was applied. Distilled water used as a template in the no-template control (NTC) was defined as the blank specimen, and 10 replicate measurements were performed on each platform. For LoD estimation, 10 candidate low-level dilution materials were first screened in duplicate to identify concentrations distributed around the expected transition zone between consistent detection and frequent non-detection. Based on this preliminary screening, five concentrations (0.5%, 0.25%, 0.0625%, 0.015625%, and 0.00390625% expected VAF) were selected for the final probit analysis because they covered the dynamic range most informative for modeling the probability of detection. Each of the five selected concentrations was then tested in eight replicates on each platform, and each replicate was classified dichotomously as positive or negative for *JAK2* V617F detection. Probit regression was subsequently performed using log-transformed expected VAF as the independent variable and hit rate as the dependent variable, in accordance with the EP17-A2 framework for molecular measurement procedures. The concentration corresponding to a 95% positive detection probability was defined as the LoD.

Precision was evaluated using three patient specimens with high, intermediate, and low *JAK2* V617F allele burdens (Pre_H, Pre_M, and Pre_L). Each level consisted of a single patient specimen without pooling, and the same specimens were evaluated by qPCR and dPCR under identical conditions. Each specimen was measured 10 times under the same conditions, and repeatability-based precision was assessed by calculating the mean, standard deviation (SD), and coefficient of variation (%CV) from the replicate values.

Linearity was evaluated using the seven-level panel (Level 1–Level 7) described above. Each prepared specimen was measured in triplicate by both qPCR and dPCR under identical experimental conditions. In accordance with CLSI EP06-A2 [22], linear regression analysis was performed to assess the relationship between expected and observed values at each level, and the coefficient of determination (R^2^) was used as the principal indicator of linearity. Linear regression analysis was conducted independently for qPCR and dPCR using the same approach, and the resulting R^2^ values and residual distributions were reviewed to determine whether linearity was maintained across the measurement range.

For LoD, linearity, precision, and method comparison analyses, exclusion was restricted to technically invalid measurements rather than statistically extreme values alone. Measurements were considered technically invalid when (i) wild-type amplification completely failed in materials where wild-type signal was expected, (ii) mutant amplification completely failed in materials expected to contain *JAK2* V617F, or (iii) visual inspection of amplification curves and fluorescence/scatter plots indicated a clear technical artifact or reaction failure. In the clinical specimen cohort used for method comparison, specimens in which either qPCR or dPCR showed complete technical failure according to these criteria were excluded from the paired analysis.

### 2.5. Comparison of qPCR and dPCR

Method comparison was performed using *JAK2* V617F VAF values measured by qPCR and dPCR in the same clinical specimens. The overall difference between the two methods was evaluated using a paired *t*-test, and the linear association between measured values was assessed by Pearson correlation analysis. Linear regression analysis was also performed to examine the relationship between quantitative results from the two assays, and Bland–Altman analysis was used to evaluate the mean difference and the 95% limits of agreement. For agreement-oriented analysis, Deming regression was additionally applied because both qPCR and dPCR are subject to measurement error, and neither assay can be regarded as error-free.

### 2.6. Clinical Data Analysis and Statistical Software

Peripheral blood test results obtained at the same time point were collected to evaluate the relationship between *JAK2* V617F VAF and white blood cell count, red blood cell count, hemoglobin, and platelet count. Correlations between each hematologic parameter and VAF were analyzed using Pearson correlation analysis, and additional between-group statistical tests were applied as appropriate to assess differences according to clinical diagnostic group or specimen characteristics. All statistical analyses were performed using Microsoft Excel 2019 (Microsoft Corp., Redmond, WA, USA) and IBM SPSS Statistics for Windows, Version 20.0.0 (IBM Corp., Armonk, NY, USA). A two-sided *p* value of <0.05 was considered statistically significant.

## 3. Results

### 3.1. Overall Quantitative Comparison of qPCR and dPCR

Quantitative results obtained by qPCR and dPCR were compared in 76 *JAK2* V617F-positive specimens. Across all specimens, the mean VAF measured by qPCR was 52.32% with an SD of 31.18%, whereas the mean VAF measured by dPCR was 55.05% with an SD of 30.88%. The measurement ranges were 1.01–95.19% for qPCR and 0.86–95.94% for dPCR, indicating broadly similar distributions across a wide concentration range for both methods (Table 2).

### 3.2. Detection Capability: Limit of Blank and Limit of Detection

**Figure 1:**
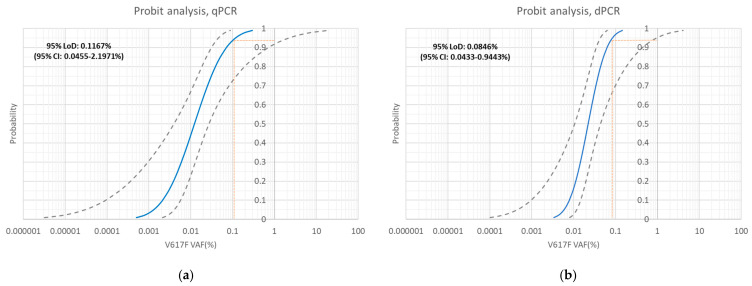
Probit plots for the 95% limit of detection (LoD) of qPCR and dPCR. (**a**) Probit regression for qPCR showing a 95% LoD of 0.1167% (95% CI, 0.0455–2.1971%). (**b**) Probit regression for dPCR showing a 95% LoD of 0.0846% (95% CI, 0.0433–0.9443%). The x-axis represents the log-transformed V617F variant allele frequency (VAF, %), and the y-axis represents the probability of detection. The gray dashed lines indicate the 95% confidence interval of the probit regression, the blue solid line represents the fitted probit regression curve, and the orange dashed line indicates the estimated LoD corresponding to a 95% detection probability. Abbreviations: qPCR, real-time quantitative PCR; dPCR, real-time digital PCR; VAF, variant allele frequency.

Detection capability was evaluated sequentially in terms of LoB and LoD according to CLSI EP17-A2. In the LoB evaluation, the final VAF values for 10 repeated NTC measurements were 0% for both qPCR and dPCR; therefore, the LoB was set at 0 for both methods. LoD evaluation was performed by probit analysis based on repeated measurements at five low-concentration levels (Supplementary Materials, Table S1). In the LoD analysis, one dPCR measurement at the lowest concentration level (LoD9) showed complete amplification failure and was treated as a technical outlier; the remaining seven replicates at this level were retained for probit modeling. Probit regression estimated the LoD corresponding to 95% detection probability as 0.1167% (95% CI, 0.0455–2.1971%) for qPCR and 0.0846% (95% CI, 0.0433–0.9443%) for dPCR, indicating a lower detection limit for dPCR than for qPCR (Figure 1).

### 3.3. Precision Analysis

**Figure 2:**
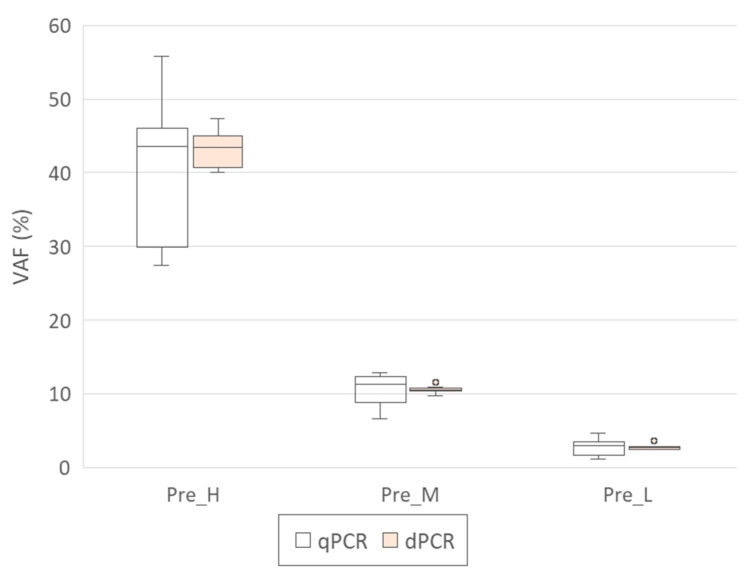
Box plots of qPCR and dPCR results from three concentration levels for precision evaluation. Box plots show the distribution of 10 replicate measurements for three *JAK2* V617F VAF levels (Pre_H, Pre_M, Pre_L) obtained by qPCR and dPCR. For the high-level sample (Pre_H), the mean VAF and coefficient of variation (%CV) were 39.64% and 22.93% by qPCR and 43.15% and 5.47% by dPCR. For the mid-level sample (Pre_M), the mean VAF and %CV were 10.55% and 20.05% by qPCR and 10.58% and 4.31% by dPCR. For the low-level sample (Pre_L), the mean VAF and %CV were 2.72% and 38.94% by qPCR and 2.71% and 12.36% by dPCR, indicating consistently lower variability for dPCR across all concentration levels, especially at low VAF. The open circles indicate individual outlier values plotted outside the whiskers of each box plot. Abbreviations: qPCR, real-time quantitative PCR; dPCR, real-time digital PCR; VAF, variant allele frequency.

Precision was evaluated by 10 repeated measurements at high, intermediate, and low concentration levels. In the high-concentration specimen, the mean VAF and %CV were 39.64% and 22.93% for qPCR and 43.15% and 5.47% for dPCR. In the precision study, one qPCR measurement for the high-concentration specimen (Pre_H) was excluded because the mutant signal was markedly overestimated compared with the other replicates, resulting in an aberrantly high VAF, whereas no technical outliers were identified for the intermediate- and low-concentration specimens. In the intermediate-concentration specimen, the mean VAFs were similar between qPCR and dPCR (10.55% and 10.58%, respectively), whereas the %CV values were 20.05% and 4.31%, respectively, indicating lower variability with dPCR. In the low-concentration specimen, the mean VAFs were again similar between qPCR and dPCR (2.72% and 2.71%, respectively), but the %CV was substantially lower for dPCR than for qPCR (12.36% vs. 38.94%), demonstrating superior repeatability of dPCR in the low-VAF range (Figure 2).

### 3.4. Linearity Assessment

**Figure 3:**
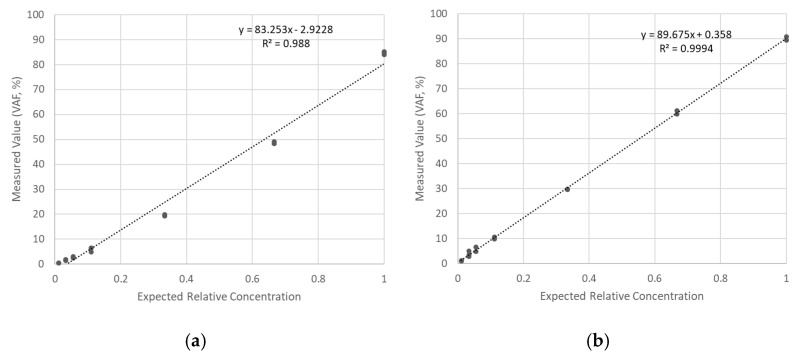
Linearity evaluation of qPCR and dPCR for measuring *JAK2* V617F variant allele frequency. In-house prepared samples with different expected *JAK2* V617F VAFs were used to evaluate linearity. (**a**) For qPCR, linear regression analysis between the expected relative concentration (RC) to the highest level (L1) and the measured VAF (%) yielded an R^2^ of 0.988. (**b**) For dPCR, the corresponding regression yielded an R^2^ of 0.999. In both panels, the x-axis represents the expected RC to L1 and the y-axis represents the measured VAF (%); black dots indicate the observed values for the seven concentration levels, and the dashed line indicates the fitted regression line, with the regression equation and R^2^ shown in the upper area of each plot. Abbreviations: qPCR, real-time quantitative PCR; dPCR, real-time digital PCR; VAF, variant allele frequency.

Linearity was evaluated using a seven-level dilution panel. Linear regression analysis between expected and observed values showed R^2^ of 0.988 for qPCR and 0.999 for dPCR, indicating good linearity across the full dilution series for both methods. However, because the dilution panel was constructed from patient specimens and the expected values were also based on measurements from the evaluated methods themselves, direct comparison of absolute accuracy is inherently limited. With this limitation in mind, relative concentration (RC) ratios were additionally compared using the highest-concentration sample (Level 1) as the reference. The expected relative ratios for Level 4 and Level 7 were 0.1111 and 0.0111 of Level 1, respectively, whereas the observed ratios were 0.0702 and 0.0056 for qPCR and 0.1216 and 0.0128 for dPCR (Table 3). Thus, qPCR showed a negative bias in RC at low concentrations, whereas dPCR did not and remained close to the expected RC values. (Figure 3). In addition, recovery percentages (measured VAF/expected VAF × 100) were calculated for each dilution level and added to Table 3. In the linearity evaluation, four dPCR measurements (one each at L1, L2, L4, and L5) were excluded because of complete wild-type amplification failure (three measurements) or simultaneous failure of wild-type and mutant amplification (one measurement). At each dilution level, at least two valid replicate measurements remained and were used for regression analysis.

### 3.5. Method Comparison Between qPCR and dPCR

**Figure 4:**
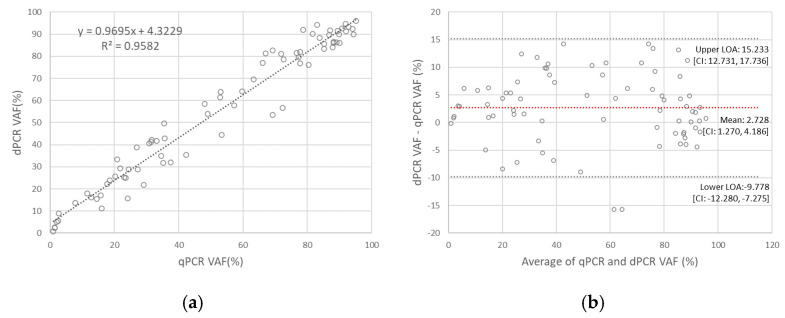
Method comparison between qPCR and dPCR for *JAK2* V617F VAF measurement. (**a**) Linear regression between *JAK2* V617F VAF (%) measured by qPCR and dPCR. Each open circle represents one clinical sample, the dashed line indicates the fitted regression line, and the regression equation and coefficient of determination (R^2^ = 0.958) are shown in the upper part of the plot. (**b**) Bland–Altman plot showing the difference between dPCR and qPCR VAF (%) against their mean. The x-axis represents the mean VAF (%) of qPCR and dPCR, and the y-axis represents the difference (dPCR − qPCR, %). The gray dashed lines indicate the upper and lower limits of agreement (LOA) with 95% confidence intervals, and the central red dashed line indicates the mean difference (−2.73 percentage points) with its 95% confidence interval. Each circle represents an individual specimen. Abbreviations: qPCR, real-time quantitative PCR; dPCR, real-time digital PCR; VAF, variant allele frequency.

Method comparison was performed using qPCR and dPCR results measured in the same clinical specimens. Among the 94 residual *JAK2* V617F-positive DNA extracts initially available, 10 specimens showed complete amplification failure by qPCR alone, 5 by dPCR alone, and 3 by both methods according to the predefined technical criteria. These 18 specimens were excluded from the paired method comparison, and the final analysis therefore included 76 clinical specimens with valid quantitative results on both platforms. Paired *t*-test analysis showed that the mean difference between qPCR and dPCR (qPCR − dPCR) was −2.73 percentage points, with a 95% confidence interval from −4.19 to −1.27 percentage points, and this difference was statistically significant (*p* = 0.00037). When the method difference (qPCR − dPCR) was examined according to specimen type, the mean differences observed for BM and PB samples were similar, and no statistically significant effect of specimen type on the qPCR−dPCR VAF difference was detected, although the limited number of BM samples warrants cautious interpretation. These findings indicate that dPCR tended to report higher VAF values than qPCR on average. Nevertheless, the Pearson correlation coefficient between the two methods was 0.9789, and linear regression analysis yielded an R^2^ value of 0.958, indicating excellent overall quantitative agreement. The regression equation was dPCR_percent = 4.32 + 0.97 × qPCR_percent, and both the intercept (*p* = 0.0034) and slope (*p* < 0.001) were statistically significant (Figure 4). In addition to ordinary least-squares regression, Deming regression was performed to account for measurement error in both assays. The Deming slope was 0.99 (95% CI, 0.942 to 1.038) and the intercept was 3.24 (95% CI, 0.329 to 6.151), with a Pearson correlation coefficient of 0.979, further confirming the near-proportional agreement between qPCR- and dPCR-based VAF values across the measurement range.

### 3.6. Association of JAK2 V617F Allele Burden with Diagnostic Groups and Hematologic Parameters

The distribution of *JAK2* V617F VAF according to diagnostic group was also evaluated (Supplementary Materials, Figure S2). The mean VAF values measured by qPCR were 33.32% in ET, 75.28% in PV, and 66.94% in PMF, while the corresponding mean VAF values measured by dPCR were 36.82%, 76.71%, and 70.38%, respectively. In comparisons between ET and PV, the VAF in the PV group was significantly higher than that in the ET group by both qPCR and dPCR (both *p* < 0.001). Because the PMF group contained only eight cases, caution is needed in interpreting direct between-group comparisons; however, both methods showed generally high VAF ranges in PMF. In an exploratory analysis, the distribution of qPCR–dPCR VAF differences across ET, PV, and PMF did not differ significantly (Kruskal–Wallis *p* = 0.453), and the corresponding box plots are provided in Supplementary Figure S2. Correlation analysis between hematologic parameters and *JAK2* V617F VAF showed a moderately strong positive correlation between qPCR-based VAF and WBC (r = 0.567, *p* < 0.001), and dPCR-based VAF also showed a significant positive correlation with WBC (r = 0.571, *p* < 0.001).

## 4. Discussion

In this study, the *JAK2* V617F allele burden was quantified using a laboratory-developed chip-based real-time digital PCR (LDT dPCR) platform established in a tertiary-care hospital clinical laboratory, and its analytical performance was systematically compared with that of a commercial qPCR assay. The two methods showed very high correlation for *JAK2* V617F VAF values (dPCR_percent = 4.32 + 0.97 × qPCR_percent; Pearson r = 0.9789), but dPCR reported higher VAF values on average (mean qPCR − dPCR difference, −2.73 percentage points) and showed superior detection capability and precision in the low-concentration range. These findings are broadly consistent with previous studies of *JAK2* V617F ddPCR and chip-based dPCR, which have reported high quantitative concordance with qPCR together with improved low-level detection performance [12,18,19].

In the detection capability analysis performed, the LoB was set at 0 for both qPCR and dPCR, and the LoD corresponding to 95% detection probability was estimated as 0.1167% for qPCR and 0.0846% for dPCR, indicating a lower detection limit for dPCR; however, these estimates should be interpreted with caution because the confidence intervals, particularly for qPCR, were relatively wide, likely reflecting the limited number of replicate measurements feasible in this laboratory-developed study and the inherent variability of very low-level dilution materials. This qPCR LoD is nearly identical to the 0.12% reported by Link-Lenczowska et al. [12], suggesting that the qPCR performance observed in the present study was comparable to that reported in the literature. In contrast, the LoD of 0.0846% observed for dPCR was somewhat higher than the ddPCR LoD of 0.01% reported by Liu et al. [20], but was essentially identical to the 0.08% LoD reported for chip-based digital PCR by Lu et al. [18]. Liu et al. [20] also reported a limit of quantification (LoQ) of 0.01% VAF for their laboratory-developed ddPCR assay and showed strong agreement with qPCR (r = 0.988). In addition, the LoD of ddPCR-based *JAK2* V617F assays has been reported to vary roughly from 0.01% to 0.1% depending on platform characteristics, DNA input, and intended clinical use, and some clinical laboratory ddPCR assays have been operated with LoD settings of 0.1–0.2% for quantitative use or 0.5% for qualitative use. Taken together, these findings suggest that although the chip-based LDT dPCR assay evaluated here may be somewhat more conservative in sensitivity than highly optimized ddPCR assays capable of ultra-low-level detection (≤0.01%), it nonetheless provides competitive low-level detection performance within the range reported for chip-based dPCR and clinical ddPCR assays targeting *JAK2* V617F [18,20].

Notably, Link-Lenczowska et al. [12] suggested that ddPCR may be advantageous for minimal residual disease (MRD) monitoring because it can track *JAK2* V617F below the LoD of qPCR. In the present study, dPCR also showed a lower LoD and superior low-level precision compared with qPCR, suggesting potential utility in treatment-response monitoring and residual disease assessment. Although the present study was based on residual diagnostic specimens and therefore requires further validation in a true MRD cohort, analytical performance capable of reliably detecting VAF values around 0.1% may still provide a realistic foundation for highly sensitive molecular follow-up in routine clinical laboratories. Beyond analytical sensitivity alone, the chip-based real-time dPCR platform evaluated here combines partition-based absolute quantification with review of real-time amplification behavior at the partition level, which may increase confidence when interpreting low-level variants and borderline signals.

In the precision study, dPCR showed lower %CV values than qPCR across the high-, intermediate-, and low-concentration ranges, with the most marked difference observed in the low-concentration specimen, where the %CV was 12.36% for dPCR and 38.94% for qPCR. These results are consistent with the technical advantages of dPCR, which uses partition-based absolute counting without reliance on an external standard curve and thereby reduces measurement variability in the low-concentration range [14,15,17,20].

Linearity assessment showed overall good linearity for both qPCR and dPCR (R^2^ = 0.988 and 0.999, respectively). However, when interpreted in terms of relative ratios to the highest concentration (Level 1), qPCR yielded lower-than-expected values in the low-concentration range, whereas dPCR maintained relative linearity closer to the expected values. These results support the possibility that dPCR may provide more consistent quantification when monitoring patients with low *JAK2* V617F burden, a feature that is particularly relevant for MRD-oriented follow-up and early detection of clonal evolution. The recovery analysis summarized in Table 3 further confirmed that qPCR tended to under-recover expected values at low dilutions, whereas chip-based dPCR maintained recoveries closer to the nominal concentrations, reinforcing the conclusion that dPCR shows less low-level bias across the linear range.

In the method comparison analysis, qPCR and dPCR showed very high quantitative agreement, with a Pearson correlation coefficient of 0.9789 and an R^2^ value of 0.958. This level of concordance is similar to the qPCR–ddPCR correlations reported by Link-Lenczowska et al. (r = 0.998) and Liu et al. (r = 0.988), indicating that the overall trend in *JAK2* V617F burden is robustly reproduced across platforms [12,20]. Thus, from the perspective of clinical quantitative comparison, the chip-based LDT dPCR assay in the present study appears to have achieved concordance comparable to that of previously reported ddPCR-based approaches.

At the same time, paired analysis showed that dPCR tended to report higher VAF values than qPCR. Although the mean difference of −2.73 percentage points between qPCR and dPCR was statistically significant, this magnitude of discrepancy is unlikely to be clinically relevant in most routine scenarios, in which therapeutic decisions and risk stratification typically rely on substantially larger changes in *JAK2* V617F allele burden. Taken together with the very high correlation and near-unity Deming slope, these findings indicate that the two methods can be regarded as broadly interchangeable across the typical clinical range, while the superior low-level detection capability and precision of chip-based dPCR may provide additional value in highly sensitive monitoring contexts such as minimal residual disease-oriented follow-up. A similar trend was described by Lu et al. [18] in a large cohort study, in which chip-based dPCR yielded higher *JAK2* V617F allele burden values than qPCR and next-generation sequencing (NGS) while better detecting low-level variants. Accordingly, the mean difference observed here is likely to reflect a structural difference between standard curve-based quantification by qPCR and partition-based absolute quantification by chip-based dPCR rather than random measurement error alone [14,15,18]. Clinically, this implies that changing analytical platforms during longitudinal follow-up may introduce a degree of systematic bias. Therefore, the same platform should ideally be maintained throughout treatment monitoring whenever possible, and regression-based calibration approaches such as that derived in the present study may be helpful when transitions between methods are unavoidable [14,18].

In the diagnostic-group analysis, both qPCR and dPCR showed higher mean VAF values in PV than in ET, while PMF also exhibited generally high VAF ranges despite the limited number of cases. These results are consistent with previous reports showing that *JAK2* V617F allele burden is associated with the clinical phenotype of MPN, and they suggest that chip-based dPCR also adequately reflects disease-specific distribution patterns [4,7,8,9]. In addition, the significant positive correlations observed between VAF and WBC by both qPCR and dPCR are in line with previous evidence that *JAK2* burden is linked to myeloproliferative activity [8,9,18].

Lu et al. [18] reported in a cohort of 283 MPN samples that chip-based dPCR detected low-burden variants more effectively and that *JAK2* V617F burden was significantly associated with WBC, LDH, and β2-microglobulin. Although the present study was smaller in scale, it is noteworthy in that it evaluated both the analytical performance and clinical associations of qPCR and chip-based LDT dPCR using real residual specimens from clinical practice. In particular, the finding that a laboratory-developed chip-based dPCR assay showed quantitative performance comparable to prior reports in actual patient specimens provides useful evidence for future standardization and clinical implementation of chip-based real-time digital PCR [17,18,19].

Moreover, because the LOAA system can operate as a single-unit assay without requiring batch-based testing, it may be more practical than many ddPCR platforms, which are generally optimized for larger test volumes, in hospitals with relatively smaller numbers of patients with hematologic malignancies who still require tumor-specific assays [23,24,25].

Taken together, this study provides integrated evidence from LoB/LoD evaluation, precision, linearity, method comparison, and analysis of clinical associations based on 76 *JAK2* V617F-positive specimens obtained from real-world clinical practice. It therefore establishes concrete analytical performance data for a chip-based LDT dPCR platform implemented in a tertiary-care hospital laboratory.

This study also has several limitations. First, this study included only residual DNA extracts from specimens that had already tested positive for *JAK2* V617F by clinically performed qualitative PCR. Therefore, the present design was suitable for quantitative method comparison and analytical performance evaluation, but it did not allow direct assessment of clinical specificity or false-positive performance in *JAK2* V617F-negative specimens. Accordingly, the findings of this study should be interpreted primarily in the context of quantitative allele burden assessment and low-level mutation monitoring, rather than as a standalone evaluation of screening performance in an unselected diagnostic population. Second, it was a retrospective single-center study with a limited sample size, and some disease subgroups, particularly PMF, contained only a small number of cases, requiring caution in interpreting between-group differences. Third, the dilution panel used for linearity evaluation was derived from patient specimens, and the expected values depended on measurements from the methods under evaluation, which limits direct comparison of absolute accuracy. Fourth, because longitudinal data for direct MRD monitoring or treatment-response follow-up were not included, the extent to which improved low-level performance translates into additional clinical benefit remains to be validated in future studies. Another major limitation of this study is that all clinical specimens were analyzed once without technical replicate measurements. This constraint reflects the limited volumes of residual diagnostic DNA available, the retrospective design using stored extracts, and the practical resource limitations of the study. As a result, the agreement statistics and Bland–Altman comparisons presented here may partly reflect within-run variability in addition to true method-related bias, and the precision of replicate-based estimates for the full clinical cohort could not be directly assessed. Future prospective multi-center validation studies that include systematic replicate measurements across the full VAF range will be essential to more fully characterize random error components and to confirm the robustness of agreement statistics derived from single-run clinical data.

## 5. Conclusions

The present study demonstrates that a chip-based LDT dPCR platform that can be established in a tertiary-care hospital laboratory has analytical performance that can meaningfully complement, and in some settings potentially replace, commercial qPCR for *JAK2* V617F quantification. Future large-scale, multicenter studies incorporating longitudinal specimens collected before and after treatment are warranted to determine the additional clinical value of chip-based dPCR-based *JAK2* V617F kinetics for prognostic prediction, molecular response assessment, and early relapse detection. In addition, extension of this approach to other driver mutations such as CALR and MPL, together with cross-platform validation and standardization, may further broaden the role of dPCR-based precision monitoring strategies in molecular diagnostics for MPNs.

## Figures and Tables

**Table 1:** Information on in-house spiked samples for performance evaluation.

Sample Name	Expected VAF (%)	Dilution Ratio	Relative Concentration to L1 (RC)
**Materials for linearity evaluation**			
L1 *	90		1
L2	60	L1:NC = 6:3	0.6667
L3	30	L1:NC = 3:6	0.3333
L4	10	L1:NC = 1:8	0.1111
L5	5	L4:NC = 5:5	0.0556
L6	3	L4:NC = 3:7	0.0333
L7	1	L4:NC = 1:9	0.0111
**Materials for LoD evaluation**			
LoD 1 (=L7)	1		
LoD 2	0.5	LoD 1:NC = 1:1	
LoD 3	0.25	LoD 2:NC = 1:1	
LoD 4	0.125	LoD 3:NC = 1:1	
LoD 5	0.0625	LoD 4:NC = 1:1	
LoD 6	0.03125	LoD 5:NC = 1:1	
LoD 7	0.015625	LoD 6:NC = 1:1	
LoD 8	0.0078125	LoD 7:NC = 1:1	
LoD 9	0.00390625	LoD 8:NC = 1:1	
LoD 10	0.001953125	LoD 9:NC = 1:1	

* In-house manufactured by properly mixing pre-measured pooled samples with a V617F mutation-negative sample. Abbreviations: LoD, limit of detection; NC, negative sample; VAF, variant allele frequency.

**Table 2:** Sample information and results of the qPCR and dPCR experiments.

		Total	ET	PV	PMF
**N**		76	40	28	8
Patient Information *					
Sex	M/F	32/38	14/25	14/11	4/2
Age	average (range)	68.44 (29–91)	67.31 (29–91)	68.92 (47–82)	73.83 (65–86)
**Sample Type (DNA extracted from)**					
BM		16	4	9	3
PB		60	36	19	5
**CBC results**					
WBC	mean (SD)	15.06 (7.6)	13.28 (7.8)	17.04 (17.04)	16.99 (11.07)
(×10^3^/uL)	range (min.–max.)	3.18–51.97	5.75–51.97	8.66–30.37	3.18–33.18
Hb.	mean (SD)	15.18 (3.48)	13.9 (2.29)	17.89 (17.89)	12.06 (4.39)
(g/dL)	range (min.–max.)	7.3–22.5	7.7–17.7	10.1–22.5	7.3–18.2
Hct.	mean (SD)	47.46 (11.4)	42.96 (7.48)	56.53 (56.53)	38.24 (14.64)
(%)	range (min.–max.)	22.8–77.2	25.9–56.8	32.5–77.2	22.8–59.1
Platelet	mean (SD)	655.74 (319.21)	800.7 (279.55)	480.46 (480.46)	544.38 (514.17)
(×10^3^/uL)	range (min.–max.)	48–1404	251–1404	157–941	48–1330
**Experiment result, VAF(%)**					
qPCR	mean (SD)	52.32 (31.18)	33.32 (27.31)	75.28 (75.28)	66.94 (24.85)
	range (min.–max.)	1.01–95.19	1.01–94.37	16.14–92.92	23.6–95.19
dPCR	mean (SD)	55.05 (30.88)	36.82 (26.94)	76.71 (76.71)	70.38 (26.95)
	range (min.–max.)	0.86–95.94	0.86–92.38	11.18–94.66	25.08–95.94

* When calculating patient information, duplicate samples from the same patient were counted as one. Abbreviations: BM, bone marrow; PB, peripheral blood; qPCR, real-time quantitative PCR; dPCR, real-time digital PCR; VAF, variant allele frequency.

**Table 3:** Measured VAF (%), relative concentrations (RC) and recovery percentage of linearity evaluation.

Sample Name	Expected VAF (%)	Expected RC to L1	Measured VAF(%), RC to L1, and Recovery(%)					
			qPCR (R^2^ = 0.988)			dPCR (R^2^ = 0.999)		
L1	90	1	84.63	1.0000	94.03	90.1	1.0000	100.11
L2	60	0.6667	48.61	0.5745	81.02	60.42	0.6706	100.70
L3	30	0.3333	19.56	0.2312	65.20	29.69	0.3295	98.97
L4	10	0.1111	5.94	0.0702	59.40	10.29	0.1142	102.90
L5	5	0.0556	2.75	0.0325	55.00	5.72	0.0635	114.40
L6	3	0.0333	1.65	0.0195	55.00	3.71	0.0412	123.67
L7	1	0.0111	0.48	0.0056	48.00	1.08	0.0120	108.00

Abbreviations: qPCR, real-time quantitative PCR; dPCR, real-time digital PCR; VAF, variant allele frequency.

## Data Availability

The data presented in this study are available on request from the corresponding author due to patient privacy and ethical restrictions.

## Supplementary Materials

The following supporting information can be downloaded at: https://www.mdpi.com/article/10.3390/diagnostics16152333/s1, Additional information for Materials and Methods; Table S1: qPCR standard curve parameters across experimental runs, including slopes, intercepts, coefficients of determination (R^2^), and amplification efficiencies for wild-type and mutant assays; Table S2: results of LoD evaluation for qPCR and dPCR, summarizing hit rates at each dilution level and probit-based LoD estimates with 95% confidence intervals; Figure S1: Output formats of the LOAA real-time digital PCR system; Figure S2: Distribution of hematologic parameters and *JAK2* V617F VAF according to disease group.
