# Supplementary material for: Comparative Evaluation of Quantitative Real-Time PCR and a Laboratory-Developed Chip-Based Real-Time Digital PCR for JAK2 V617F Allele Burden in Myeloproliferative Neoplasms
Source: Diagnostics (Basel). 2026 Jul 25;16(15):2333. doi: 10.3390/diagnostics16152333 (PMC13465721; doi:10.3390/diagnostics16152333)
Supplement: Supplementary file 1 [file diagnostics-16-02333-s001.zip › diagnostics-4404185-supplementary.pdf]

Supplementary Materials

Comparative Evaluation of Quantitative Real-Time PCR and a Laboratory-Developed Chip-Based Real-Time Digital PCR for *JAK2* V617F Allele Burden in Myeloproliferative Neoplasms

Materials and Methods

- Quantitative Real-Time PCR for JAK2 V617F

Quantitative analysis of JAK2 V617F was performed using the Bio-Rad CFX96 real-time PCR system (Bio-Rad Laboratories, Hercules, CA, USA) and the Real-Q JAK2 V617F Quantification Kit (Biosewoom, Seoul, Korea). All experimental procedures were conducted according to the manufacturer's instructions for use (IFU). The assay was performed in a 96-well plate format, with wild-type (WT) DNA and JAK2 V617F mutant DNA measured independently in separate wells. Fluorescence signals were detected in a single FAM channel.

Sample DNA concentrations were initially measured using a NanoDrop spectrophotometer and then normalized to 5 ng/µL genomic DNA by dilution with distilled water. Five microliters of each sample DNA were added to each reaction, corresponding to 25 ng of genomic DNA per reaction. The PCR reaction mixture consisted of 5 µL of sample DNA and 20 µL of master mix, yielding a total reaction volume of 25 µL. The master mix contained 12.5 µL of 2X PCR reaction mixture, 4 µL of probe and primer mixture, and 3.5 µL of water. Five microliters of each standard material were also added per reaction, and a no-template control (NTC) consisting of 5 µL of distilled water instead of DNA was included in every run.

For absolute quantification, four plasmid DNA standards included in the kit were run in parallel in each experiment. The standard concentrations were 2×10^6, 2×10^4, 2×10^3, and 2×10^2 copies, and separate standard curves were generated for the WT and mutant assays. Cycle threshold (Ct) values obtained from the standards were used to construct standard curves, from which the Ct values of the samples were converted into absolute copy numbers (Table S1.). PCR cycling conditions were as follows: one cycle at 50°C for 2 min, one cycle at 95°C for 10 min, followed by 45 cycles of 95°C for 15 s and 62°C for 45 s. FAM fluorescence was measured during the 62°C step of each cycle. All samples and standards were analyzed in a single measurement without duplicate testing. VAF was calculated using copy numbers derived from the WT and mutant reactions according to the following formula: VAF (%) = mutation copies / (mutation copies + wild type copies) x 100

Table S1. qPCR standard curve parameters across experimental runs

Run date	Lot No.	Target	No. of points	Slope	Intercept	R^2	Efficiency (%)
2024-09-02	531T-017	WT	4	-3.17	42.00	0.9996	106.7
		MUT	4	-3.10	41.80	0.9975	110.0
2024-09-09	531T-018	WT	4	-3.22	41.92	0.9999	104.3
		MUT	4	-3.36	43.34	0.9996	98.2
2024-09-25	531T-018	WT	4	-3.12	40.94	0.9988	109.4
		MUT	4	-3.13	41.92	0.9966	108.5
2025-02-14	531T-018	WT	4	-3.09	42.05	0.9974	110.9
		MUT	4	-3.34	43.28	0.9999	99.1
2025-02-17	531T-018	WT	4	-3.23	42.89	0.9994	103.8
		MUT*	3	-3.03	42.30	0.9983	113.6
Overall (mean)		WT		-3.17	41.96	0.9990	107.0
		MUT		-3.20	42.53	0.9984	105.9

* One of V617F material (2×10^2) showed no amplification signal.

- Chip-Based Real-Time Digital PCR Assay for JAK2 V617F

The LDT dPCR assay was performed using the LOAA system (Optolane, Seongnam, Korea), a chip-based real-time digital PCR platform. This instrument is a cartridge-based modular system in which cartridges operate independently, allowing the number of simultaneously running cartridges to be adjusted according to the purpose of the experiment and the required throughput. In the present study, a configuration capable of analyzing up to eight cartridges simultaneously was used. Each reaction was performed using a blank Dr.PCR 20K Cartridge-PF (Cat. No. CTR002-R; Optolane) for research use only (RUO), into which the investigator-prepared reaction mixture and sample were loaded.

Primers for JAK2 V617F detection were designed to generate a 92-bp amplicon including the c.1849G(V617) position based on the JAK2 reference sequence NM_001322194.2. The forward primer (primer_F) sequence was 5′-AAGCTTTCTCACAAGCATTTGGTTT-3′, and the reverse primer (primer_R) sequence was 5′-AGAAAGGCATTAGAAAGCCTGTAGTT-3′. Probes were designed to specifically recognize the wild-type sequence and the c.1849G>T (V617F) mutant sequence at position c.1849. The wild-type probe (probe_WT) sequence was 5′-/56-FAM/TCTCCACAGACACATAC/3MGBSFCQ1/-3′, and the mutant probe (probe_MUT) sequence was 5′-/5SFC-C610/TCCACAGAAACATAC/3MGBSFCQ2/-3′. Fluorescence channels were 56-FAM (green) for the wild-type probe and 5SFC-C610 (red) for the mutant probe.

Dr.PCR(TM) Onestep 2X dRT-PCR mixture (Optolane), a dedicated mastermix, was used for the reactions. Patient DNA samples were quantified by NanoDrop spectrophotometry and normalized to 5 ng/μL, as described for qPCR. For a final cartridge input volume of 30 μL, each reaction contained 1.0 μL primer_F (15 μM), 1.0 μL primer_R (15 μM), 0.5 μL probe_WT (15 μM), 1.0 μL probe_MUT (15 μM), 15.0 μL Dr.PCR Onestep 2X dRT-PCR mixture, 6.5 μL distilled water, and 5.0 μL template DNA. Each reaction contained a total of 25 ng genomic DNA, as same amount used in qPCR experiment.

PCR cycling conditions were set according to the manufacturer's recommendations for the Optolane instrument and cartridge. UDG treatment was performed at 50°C for 3 min for one cycle, followed by pre-denaturation at 95°C for 15 min for one cycle. Amplification consisted of 50 cycles of denaturation at 95°C for 10 s and annealing/extension at 58°C for 30 s. Ramp rates were set at 15°C/s for the UDG treatment and pre-denaturation steps and 10°C/s for the amplification cycles.

After amplification, raw data generated by the LOAA system were transferred to a PC and analyzed using OnPointPro software version 1.1.9 (Optolane). For each reaction, fluorescence scattergrams and real-time amplification curves were reviewed to confirm that partition-level positive/negative calling had been appropriately performed in both the wild-type and mutant channels. The software performed absolute quantification by accounting for the fraction corresponding to 5 μL of template DNA (25 ng genomic DNA) within the final 30 μL cartridge reaction volume and reported copy numbers for wild-type and mutant targets separately. In this study, the final copy numbers reported by the software were used without further adjustment. VAF was calculated in the same manner as for qPCR. All specimens were analyzed once without replicate testing.

Results

3.2. Detection Capability: Limit of Blank and Limit of Detection

The selection of concentrates and results of LoD evalution was described in Table S2.

Table S2. Results of LoD evaluation

Name	Expected VAF	Positive Count in screening test*	Positive in evaluation test**					
			qPCR			dPCR		
			Total Count	Positive Count	Hit rate (%)	Total Count	Positive Count	Hit rate (%)
LoD1	1%	2***	-	-	-	-	-	-
LoD2	0.50 %	2	8	8	100	8	8	100
LoD3	0.25 %	2	8	8	100	8	8	100
LoD4	0.125%	2	-	-	-	-	-	-
LoD5	0.0625 %	2	8	7	87.5	8	7	87.5
LoD6	0.03125%	1	-	-	-	-	-	-
LoD7	0.015625 %	0	8	4	50	8	3	37.5
LoD8	0.0078125%	1	-	-	-	-	-	-
LoD9	0.00390625 %	0	8	2	25	7****	0	0
LoD10	0.001953125%	0	-	-	-	-	-	-

* Screening tests for each material were performed in duplicate using digital PCR.

** LoD evaluation experiments were conducted independently; results from the initial screening tests were not included in the final LoD analysis.

*** In the screening test, LoD1 showed amplification failure in both replicate measurements; therefore, the results for L7 (the same concentration level) from the linearity evaluation were used instead.

**** One dPCR measurement at the LoD9 level showed complete amplification failure and was considered a technical outlier; this result was excluded from the final evaluation.

(A)

Ch01: FAM

Well Information		Dr. PCR Results		Abs Quantification	
Well Volume	0.936 nl	Valid wells	17717	Target name	Test
Template Volume	10 µl	Invalid wells	2446	Threshold	434.31
Mixture Volume	20 µl	Positive wells	1457	Concentration	275.05 C/µl
Sample Volume	0 µl	Negative wells	16260	95% CI Lower	260.93 C/µl
Elution Volume	0 µl	Positive Ratio(%)	8.22	95% CI Upper	289.18 C/µl

Ch02: chamel610

Well Information		Dr. PCR Results		Abs Quantification	
Well Volume	0.936 nl	Valid wells	17717	Target name	Test2
Template Volume	10 µl	Invalid wells	2446	Threshold	492.971
Mixture Volume	20 µl	Positive wells	2049	Concentration	393.92 C/µl
Sample Volume	0 µl	Negative wells	15668	95% CI Lower	376.86 C/µl
Elution Volume	0 µl	Positive Ratio(%)	11.57	95% CI Upper	410.99 C/µl

Ch01 : Ch02 Ratio		Color	Sample ID
Ch01 (+) Ch02 (+)	133	● (blue)	6
Ch01 (+) Ch02 (-)	1324	● (green)	
Ch01 (-) Ch02 (+)	1916	● (red)	
Ch01 (-) Ch02 (-)	14344	● (yellow)	
Ratio (%)	69.82		

Scatter Chart

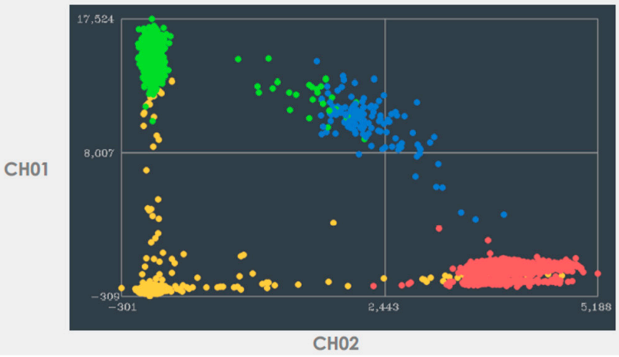

(B)

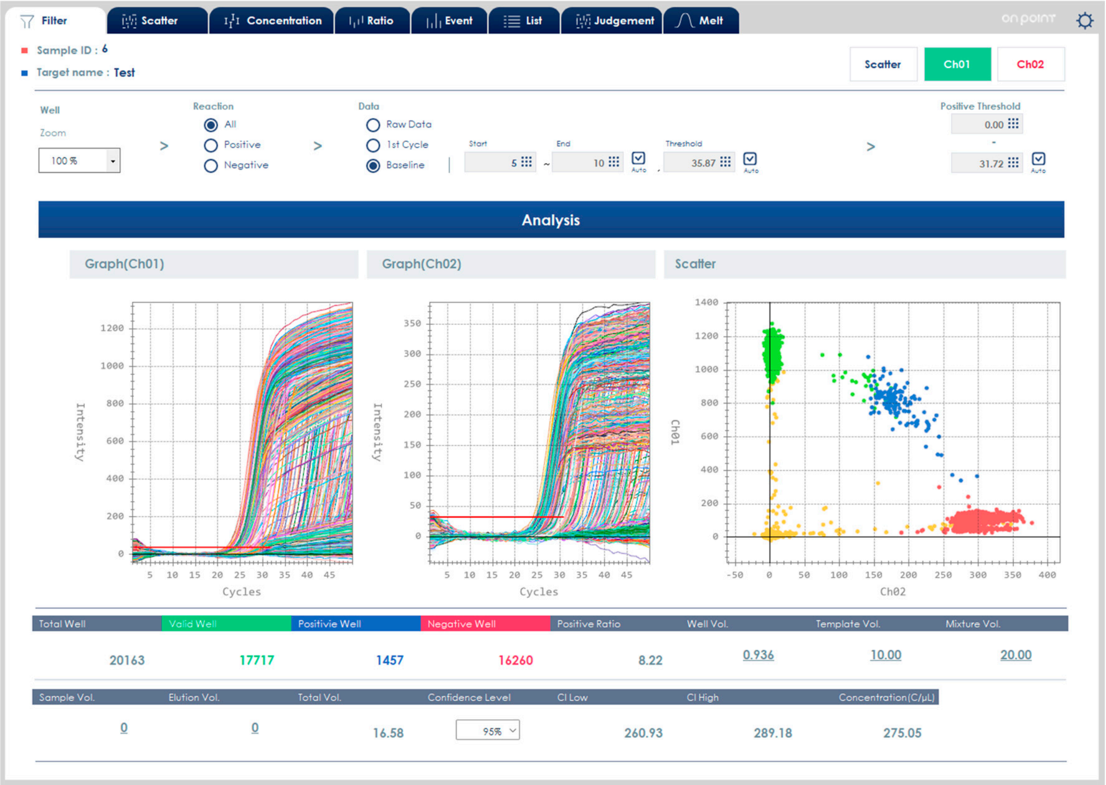

Figure S1. Output formats of the LOAA real-time digital PCR system.

(A) Example of the result report generated by the LOAA analyzer for the laboratory-developed test (LDT) JAK2 V617F quantitative digital PCR assay. The report displays partition-level fluorescence results as scattergrams for channel 1 (Ch01, FAM) and channel 2 (Ch02, Chamel610/Cal Fluor Red 610), and provides the absolute copy numbers and 95% confidence intervals calculated from the proportion of positive partitions in each channel in a PDF format.

(B) Screenshot of the RUO analysis software OnPointPro version 1.1.9 (Optolane) after loading a raw data file (.odpi2) and selecting the Ch01 tab, in which detailed information for Ch01 (FAM) is shown in the lower panel. The software presents amplification curves for each partition together with channel-specific scattergrams, enabling visual inspection of amplification quality. Thresholds and other analysis parameters can be adjusted as needed, and specific regions of interest within the scattergram can be selected for focused review.

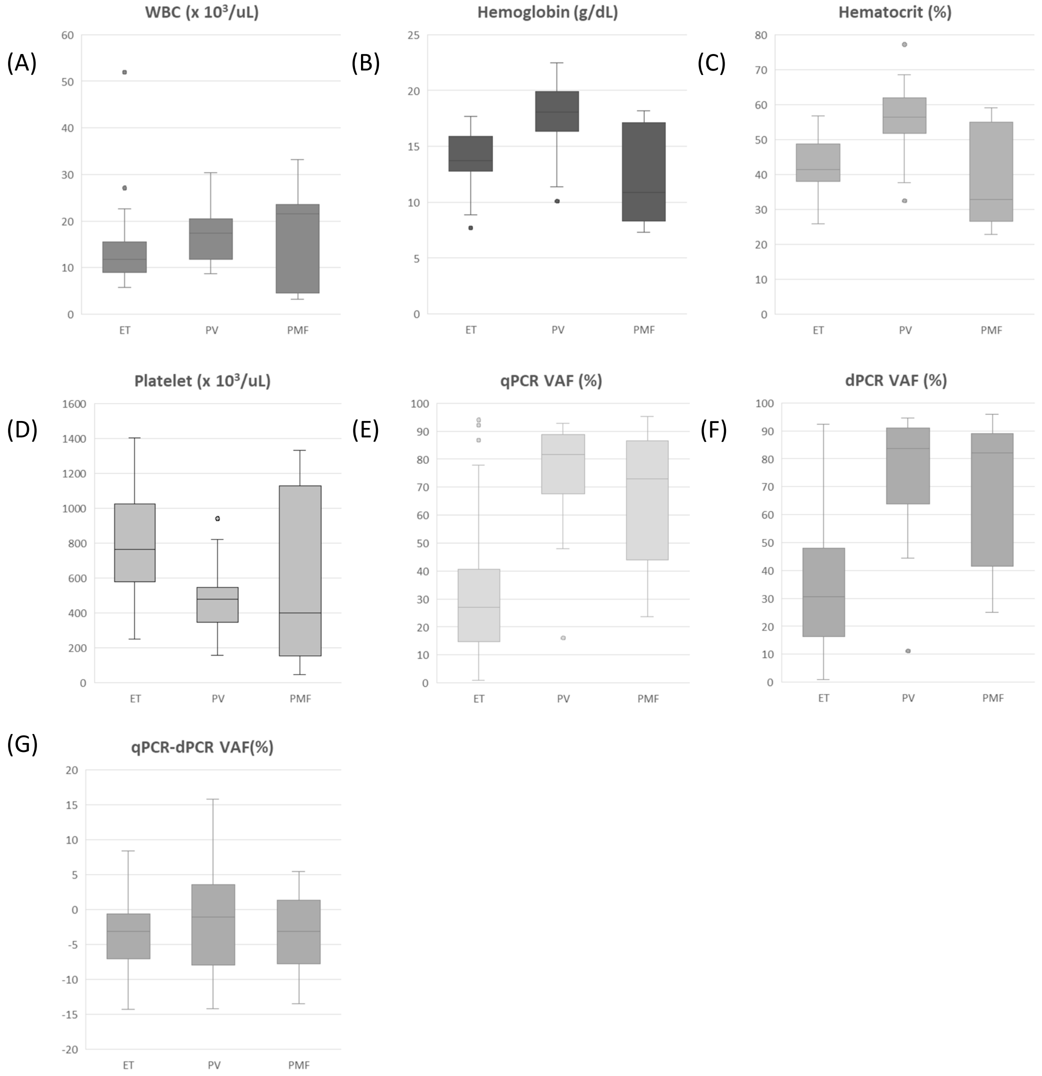

Figure S2. Distribution of hematologic parameters and JAK2 V617F VAF according to disease group.

Box plots show the distribution of hematologic parameters and JAK2 V617F variant allele frequency (VAF) according to disease group: essential thrombocythemia (ET, n = 40), polycythemia vera (PV, n = 28), and primary myelofibrosis (PMF, n = 8). Panels represent white blood cell count (WBC, $\times 10^3$/μL) **(A)**, hemoglobin (g/dL) **(B)**, hematocrit (%) **(C)**, platelet count ($\times 10^3$/μL) **(D)**, qPCR-based VAF (%) **(E)**, dPCR-based VAF (%) **(F)**, qPCR-dPCR VAF (%) (difference) **(G)**. For each parameter, the central line indicates the median, the box the interquartile range (IQR), and the whiskers the range of non-outlier values; individual outliers, if present, are shown as separate points.
